# The Microbiome Tumor Axis: How the Microbiome Could Contribute to Clonal Heterogeneity and Disease Outcome in Pancreatic Cancer

**DOI:** 10.3389/fonc.2021.740606

**Published:** 2021-09-23

**Authors:** Meghna Basu, Lisa-Marie Philipp, John F. Baines, Susanne Sebens

**Affiliations:** ^1^ Max Planck Institute for Evolutionary Biology, Plön, Germany; ^2^ Section of Evolutionary Medicine, Institute of Experimental Medicine, Kiel University, Kiel, Germany; ^3^ Institute for Experimental Cancer Research, University Hospital Schleswig-Holstein (UKSH) Campus Kiel, Kiel University, Kiel, Germany

**Keywords:** PDAC, microbiome, CSC, microbiome-targeted therapy, drug resistance, tumor heterogeneity, cancer stemness

## Abstract

Pancreatic ductal adenocarcinoma (PDAC) is one of the most malignant cancers. It is characterized by a poor prognosis with a 5-year survival rate of only around 10% and an ongoing increase in death rate. Due to the lack of early and specific symptoms, most patients are diagnosed at an advanced or even metastasized stage, essentially limiting curative treatment options. However, even curative resection of the primary tumor and adjuvant therapy often fails to provide a long-term survival benefit. One reason for this dismal situation can be seen in the evolution of therapy resistances. Furthermore, PDAC is characterized by high intratumor heterogeneity, pointing towards an abundance of cancer stem cells (CSCs), which are regarded as essential for tumor initiation and drug resistance. Additionally, it was shown that the gut microbiome is altered in PDAC patients, promotes Epithelial-Mesenchymal-Transition (EMT), determines responses towards chemotherapy, and affects survival in PDAC patients. Given the established links between CSCs and EMT as well as drug resistance, and the emerging role of the microbiome in PDAC, we postulate that the composition of the microbiome of PDAC patients is a critical determinant for the abundance and plasticity of CSC populations and thus tumor heterogeneity in PDAC. Unravelling this complex interplay might pave the way for novel treatment strategies.

## Introduction

Pancreatic ductal adenocarcinoma (PDAC) is one of the most common lethal cancer entities with hardly 10% of the patients surviving up to 5 years after diagnosis (1). Owing to the lack of early and specific symptoms, the majority of patients are diagnosed at an advanced- or even metastasized stage (2). This also implies that only 20% of the patients are eligible for resection of the primary tumor combined with adjuvant chemotherapy. However, in most cases even this curative treatment regimen only provides a temporary survival benefit, due to relapse or the development of metastases during or shortly after therapy. One reason for this poor prognosis can be seen in the evolution of resistances towards therapeutic drugs, e.g. through the activation of multidrug resistance and pro-survival pathways (3–5). Furthermore, PDAC is characterized by a pronounced inflammatory tumor stroma, which besides genetic and epigenetic alterations also contributes to the acquisition of a drug resistant phenotype in PDAC cells (6, 7).

The emergence of chemoresistance has been linked to Epithelial-Mesenchymal-Transition (EMT) in diverse cancer entities, including PDAC (8–11). Primarily, EMT is regarded as a key process in metastasis by which epithelial tumor cells acquire the capability to disconnect from the primary context and disseminate to secondary sites. Since EMT can also be seen as a dedifferentiation process, it is not surprising that EMT has been associated with the acquisition of cancer stem cell (CSC) properties. Due to their self-renewal potential and ability to undergo asymmetric cell division, CSCs are undifferentiated cells that are essential for tumor initiation and the emergence of more differentiated cell clones within the tumor. Thus, intratumor heterogeneity of PDAC might be another determinant for the response to therapeutic drugs, as particularly CSCs are highly resistant to cancer therapies (12–16).

As outlined in the review by Zhang et al. recently published in Frontiers in Oncology, the gut and tumor microbiome have emerged as a promising therapeutic target for PDAC (8, 17, 18), due to its impact on tumorigenesis and drug resistance in PDAC (19, 20). Several studies in PDAC patients demonstrated important links between the patient`s tumor microbiome and disease progression, such as correlations between patient survival and tumor microbiome diversity (21) or facilitating immune suppression (19). These findings support a link between the microbiome, disease progression and outcome of PDAC patients. Moreover, chronic inflammation associated with long-term microbial infection promotes EMT, which in turn contributes to drug resistance, cancer progression and metastasis (summarized in 8). Since EMT is linked to the acquisition of CSC properties, we postulate that the abundance and plasticity of CSCs, and thereby intratumor heterogeneity in PDAC, are critically modulated by the patient`s microbiome (of different body compartments). Considering this possible association might provide the basis for innovative therapeutic strategies targeting the microbiome.

## Epithelial-Mesenchymal-Transition

EMT is regarded as a prerequisite for epithelial/carcinoma cells to disseminate from the primary tumor to secondary sites. Undergoing this process implies a loss of typical epithelial characteristics and a gain of mesenchymal properties, causing a fundamental functional switch from stationary to a more motile and invasive phenotype. In detail, expression of epithelial proteins like E-cadherin or occludin, both being important for epithelial cell-cell contacts, are diminished, while expression of mesenchymal markers such as N-cadherin, Vimentin, L1CAM or the transcription factor Zeb1 are enhanced (22). Accordingly, EMT is a process by which cells lose their original differentiation and function, which can be regained at secondary sites by reversion of EMT, a process called Mesenchymal-Epithelial-Transition (MET). Thus, it is not surprising that EMT coincides with the acquisition of CSC-characteristics in tumor cells (23–25). Mani et al. demonstrated that breast cancer cells that have undergone EMT acquire a stem cell-like phenotype, and subsequently these stem cell-like cells resemble cells that have undergone EMT (25).

## Cancer Stem Cells

Similar to physiological stem cells, CSCs are characterized by the ability to proliferate indefinitely and to divide asymmetrically, giving rise to both stem cells and differentiated short-lived daughter cells with limited proliferative capability (26–29). Based on these properties, CSCs - although accounting only for a small part of the entire tumor cell population - are regarded as essential for tumor initiation and progression as well as for tumor heterogeneity (27, 30–32). According to the current model, CSCs are not a fixed cell population, but that the aforementioned characteristics can be acquired and lost dependent on environmental stimuli, as CSC are highly dependent on their niche, i.e. oxygen level, surrounding stromal cells and their released factors (24, 29, 33–36). Hence, factors like oxidative, inflammatory and nutritional stress, to which tumor cells are commonly subjected to, determine the differentiation of non-CSCs into CSCs and *vice versa*. From an evolutionary point of view, this model implies that changes in the tissue microenvironment (e.g., inflammation and/or microbiome changes) lead to the selection of subpopulations of CSCs in a Darwinian manner. As a consequence, these CSCs develop strategies that enable them to survive the adverse conditions of the host (37). This might also provide an explanation for the marked resistance of CSCs to different therapies (8). For instance, chemotherapies aim to decrease the total number of rapidly proliferating tumor cells. However, since CSCs rarely divide and exhibit high levels of drug export molecules, this is only partially successful, as the main tumor cell population might be removed while CSCs survive and can give rise to recurrences or metastases (30, 38–42).

In summary, CSCs contribute to tumorigenicity, tumor progression, metastasis, recurrence as well as therapy resistance in PDAC (26, 42, 43). Given the fact that EMT as well as the interconversion from non-CSC to CSC are both processes defining tumor cell plasticity and heterogeneity, and either process is highly dependent on the inflammatory/stress level of the surrounding microenvironment, it is reasonable to postulate that the microbiome is another important determinant for defining evolution of CSC. Confirming the contribution of the microbiome to tumor cell plasticity might provide additional mechanistic insight into tumorigenesis and the survival of PDAC patients.

## The Microbiome - PDAC Axis

### Alterations of the Microbiome in PDAC Patients

The human gut microbiota is comprised of a collection of different bacteria, archaea, fungi, viruses and protozoa. Its composition is unique to each individual and is influenced by a variety of environmental factors such as the mode of birth, age, diet, and, disease (44, 45). The microbiome plays vital roles in immune development, nutrition, energy metabolism and host defense (45). Generally, a higher bacterial diversity is characteristic of a healthy gut microbiome, whereas low diversity accompanies diseases such as inflammatory bowel disease, diabetes mellitus type 2, asthma and various cancers (46–50). An inflammatory environment favors pro-inflammatory bacteria in the diseased gut, thereby establishing a cycle of inflammation (51). In some cases of *Enterococcus faecalis* infection, the bacterium infiltrates the patient´s pancreas and initiates inflammation, resulting in the progression of chronic pancreatitis (52). A state of chronic inflammation as manifested e.g. in chronic pancreatitis or *Helicobacter pylori* infection in the gut is a known risk factor of PDAC development (53–55). Several routes by which bacteria can migrate into the PDAC microenvironment have been proposed, such as through the bile duct, portal circulation system or mesenteric lymph nodes (8). A number of studies support these routes, for example microbiome analysis of the cyst fluid of intraductal papillary mucinous neoplasms (IPMNs) with high-grade dysplasia revealed the presence of *Fusobacterium nucleatum* and *Granulicatella adiacens*, which are commonly found in the oral cavity (56, 57). In line with these findings, Mitsuhashi et al. identified *Fusobacterium* species being enhanced in tumor tissues of PDAC patients and associated with a worse prognosis (58).

The study by Geller et al. revealed that most bacterial species that were identified by 16S rRNA gene sequencing in PDAC tissues belong to Gammaproteobacteria and are predominantly members of the *Enterobacteriaceae* and *Pseudomonadacea* families (17). Furthermore, pancreas, bile, and jejunum samples from patients undergoing pancreaticoduodenectomy showed a distinctly different microbiome than healthy controls (59). Although the process of bacterial translocation from the oral cavity and gut into the pancreatic (tumor) tissue is not fully understood, we can speculate on the factors and mechanisms that favor this migration. For example, the formation of a new niche that offers lower colonization resistance and provides nutrition in the form of increased glycan levels might favor the migration of bacteria into the tumor microenvironment (60). In line with this hypothesis, the tumor microenvironment is enriched with structural proteins, proteoglycans, adapter proteins and enzymes, as well as tumor associated inflammatory cells such as myofibroblasts or macrophages, which are known producers of the aforementioned factors (61). Together, these changes in the microenvironment provide advantageous conditions that may facilitate bacterial migration from the gut into the pancreas on the one hand, and promote tumor development and progression on the other hand.

### Impact of an Altered Microbiome on EMT and Therapy Resistance

It was demonstrated that an inflammatory tumor microenvironment and tumor associated microbiome can promote EMT by inducing various signaling pathways that lead to the activation of different EMT transcription factors. Thus, it could be shown that infections by certain pathogens such as *F. nucleatum* are able to induce phosphorylation, and thus internalization of the epithelial marker protein E-cadherin. This in turn mediates the release of bound β-catenin, which translocates into the nucleus and influences the expression of EMT related genes. As a consequence, tumor cells undergo EMT and become capable of leaving the primary tumor and disseminate to secondary sites (8, 54, 55, 62). Given the fact that *Fusobacteria* species are already enriched in premalignant lesions such as IPMN, and their abundance in PDAC tissues is associated with a worse outcome (56, 58), it seems plausible that their abundance contributes to PDAC progression by EMT induction. Importantly, a distinct tumor microbiome was shown to clearly discriminate long-term survivor (LTS) from short-term survivor (STS) PDAC patients. Performing taxonomic profiling of bacterial DNA from 36 LTS and 32 STS PDAC patients revealed a higher species diversity in tumor samples of LTS patients associated with a significantly longer overall survival (median survival: 9.66 years) compared to STS patients with a low diversity (median survival: 1.66 years) (21). Overall, these findings strongly support a tumor promoting role of the microbiome and its suitability as a potential therapeutic target. This view is further supported by recent studies indicating that microbes residing in the tumor microenvironment can contribute to drug resistance, which is a major problem in PDAC treatment. In detail, Geller et al. (17) identified that the tumor microbiome of PDAC patients shows a high abundance of bacterial species belonging to the class Gammaproteobacteria. These bacteria express the enzyme cytidine deaminase (CDD) predominantly in its long form, which enables the metabolization of the chemotherapeutic drug gemcitabine (2′,2′-difluorodeoxycytidine), which is commonly used for treatment of PDAC patients in the adjuvant and palliative setting, into its inactive form (2′,2′-difluorodeoxyuridine) (17).

Besides demonstrating a novel tumor promoting role of microbiota, these findings suggest a potential mutualistic relationship between tumor cells and bacteria, with both of them exhibiting a form of parasitism towards the host. Furthermore, it can be postulated that the presence of a distinct microbiome provides favorable conditions for selection and survival of those tumor cell clones that have evolved the best survival strategies and exhibit a high degree of plasticity, such as CSCs. Enrichment and survival of CSCs within the tumor essentially add to PDAC development and progression on the one hand, and therapy resistance on the other hand.

### First Approaches Towards Microbiome Targeted Therapy

Therapy resistance, e.g. against cytostatic drugs, but also immunotherapies such as cytotoxic T-lymphocyte-associated protein 4 (CTLA-4) inhibitors, is still a major clinical challenge in the treatment of PDAC patients, and has been related to tumor heterogeneity implying the presence of CSCs (12–16). As outlined above, evidence supporting a tumor-promoting role of an altered host microbiome at different sites is accumulating. Pathological microbiome alterations apparently contribute to tumor development and progression in different ways, e.g. by shaping host immunity, impacting differentiation processes such as EMT and determining the efficacy of PDAC therapy (17–19).

Preclinical studies already strongly support the concept of modulating the host`s microbiome to improve treatment responses in PDAC, whereby antibiotic-treated mice displayed a marked anti-tumor response to gemcitabine compared to the control mice, which exhibited rapid tumor progression. Additionally, histological analysis of tumor tissues revealed more apoptosis induction in tumor cells when gemcitabine was applied in combination with antibiotics compared to gemcitabine monotherapy (17).

Furthermore, fecal microbiota transplant (FMT) has gained attention as a promising anti-tumor therapy (21). Thus, an increase in tumor growth was observed in mice after FMT from STS PDAC patients compared to that from LTS PDAC patients (21). These findings correlated with the microbiome composition and overall survival times of these patients, and indicate that the transplanted microbiome from STS patients promotes tumor growth, while that from LTS PDAC patients displays the opposite effect, leading to a slower tumor growth compared to the control group without FMT (21). Furthermore, this study revealed a strong correlation between microbiome diversity and elevated numbers of CD3+, CD8+ and Granzyme B+ T cells in tumor tissues of LTS PDAC patients compared to STS patients. These results support the view that the tumor microbiome modulates immunity in the tumor microenvironment, and thus influences the dynamic interplay between tumor and immune cells during tumorigenesis. In this context, a preclinical study showed that the efficacy of the CTLA-4 inhibitor Ipilimumab is increased in the presence of the gut commensal *Bacteroides* spp., which could in turn be reverted upon administration of antibiotics (18). The presence of these commensals affects interleukin-12 dependent T helper-1 immune responses, which in turn modulates tumor control in mice and humans while preserving intestinal integrity. These findings thus point toward a role of gut commensals in shaping the host immune response and thereby controlling tumor growth. Overall, these findings indicate that the composition of the tumor- as well as the gut microbiome are essential determinants of PDAC evolution and therapeutic responses (17, 18, 20). **Table 1** lists recent studies that have found tangible associations between disease progression and immune regulation with the host microbiome composition. As already mentioned above some of these studies have even singled out distinct groups of bacteria that influenced these changes. Naturally, clinical trials focusing on compiling 16S rRNA profiles of PDAC patient samples are on the rise (based on http://clinicaltrials.gov/). There is mounting evidence that patient microbiome composition can be used as a biomarker for disease progression as well as to increase therapeutic efficacy of PDAC treatment (**Table 1**). Likewise, Leinwand & Miller propose selectively tailoring PDAC therapy with respect to the patients’ intratumoral and gut microbiome to enhance therapeutic efficacy (66).

**Table 1:** Compilation of studies on the impact of the microbiome in cancer progression and drug resistance as well as its potential as a biomarker or therapeutic target.

Study system	Targeted Pathway/Treatment	Specific Microbiome	Biomarker/Target Potential	Reference
MCA205 sarcomas in mice housed in specific pathogen–free (SPF) versus germ-free (GF) conditions	CTLA-4	*Bacteroides thetaiotaomicron* or *Bacteroides fragilis*	Ipilimumab in presence of *Bacteroides* spp. Increases response to CTLA4 blockade. Detection of *Bacteroides* spp. as predictive biomarker CTLA4 inhibition therapy	(18)
Subcutaneous B16.SIY melanoma in C57BL/6 mice with different microbiomes	Programmed cell death protein 1 ligand 1 (PD-L1)	*Bifidobacterium*	(PD-L1)–specific antibody therapy in combination with oral Bifidobacterium administration exerts anti-tumor effect. Detection of oral *Bifidobacterium* as predictive biomarker	(63)
Subcutaneous colon carcinoma (MC-26) in BALB/c mice	Nucleoside analogues- gemcitabine	Bacteria expressing long isoform of bacterial enzyme cytidine deaminase (CDD) e.g.: Gammaproteobacteria, & *M. hyorhinis which expresses the* (short isoform) renders gemcitabine ineffective.	Gemcitabine in combination with ciprofloxacin increases the antitumor response; Detection of CDD and bacteria as a predictive biomarker for gemcitabine treatment	(17)
Formalin-fixed, paraffin-embedded (FFPE) patient tissue specimens of PDAC patients	NA	Fusobacterium species positively correlated with worse prognosis	Detection of Fusobacterium species in PDAC tissue as a prognostic biomarker	(58)
Bacterial DNA from surgically resected (LTS & STS) patient PDAC tumors	NA	LTS patients were enriched in Proteobacteria (Pseudoxanthomonas) and Actinobacteria (Saccharopolyspora and Streptomyces)	Detection of Pseudoxanthomonas, Saccharopolyspora and Streptomyces as a prognostic biomarker	(21)
Orthotopically implanted KPC PDAC cell lines in antibiotic- treated C57BL/6 mice	NA	FMT from LTS patient stool samples inhibited tumor growth	FMT after antibiotic treatment can be used as anti-tumor therapy.	(21)
Cyst fluid and peripheral blood liquid biopsies from patients with suspected pancreatic cystic lesions	NA	Intracystic bacterial DNA quantity positively correlated with the neoplastic grade severity of IPMN, like *G. adiacens, F. nucleatum, P. micra, E. corrodens, H. parahaemolyticus, A. odontolyticus, P. melaninogenica* and *Campylobacter* spp.	Detection and abundance of these bacteria as a diagnostic biomarker	(56)
Pancreatic juice and bile from PDAC and CP patients; caerulein-injected mice model for CP	NA	*Enterobacter* and *Enterococcus spp* were detected in pancreatic tissue and bile from PDAC and CP patients and in CP mice but not in controls suggesting these bacteria may be involved in CP and PDAC development	Detection of *Enterobacter* and *Enterococcus spp may serve* as a diagnostic/prognostic biomarker i	(52)
Antibiotic treated C57Bl/6 mice inoculated with EL4 lymphoma, MC38 colon carcinoma, or B16 melanoma cells	Antitumor immune responses	Antibiotic treated mice showed impaired therapy efficacy and resulted in lower cytokine production and tumor necrosis after CpG-oligonucleotide based immunotherapy.	Ensuring the presence of an intact gut microbiome prior to therapy may boost treatment efficacy	(20)
Germ-free mice transplanted with responder fecal material and later inoculated with B16.SIY melanoma cells	Programmed cell death protein 1 ligand 1 (PD-L1)	*Bifidobacterium longum, Collinsella aerofaciens*, and *Enterococcus faecium were abundantly found in the microbiome of the responders to a*nti-PD-1-based immunotherapy	Possible supplementation of probiotic cocktails containing beneficial bacteria may increase anti–PD-1 antibody efficacy	(64)
KRAS^G12D^ TP53^R172H^Pdx-Cre (KPC) mice	NA	Distinct microbial dysbiosis was observed with PDAC tumor growth	Microbiome profiling can serve as a prognostic marker for disease progression	(65)
KRAS^G12D^ TP53^R172H^Pdx-Cre (KPC) mice and fecal samples of PDAC patients	Innate and adaptive immune cell signaling	*Proteobacteria, Actinobacteria, Fusobacteria*, and *Verrucomicrobia* was enriched in the pancreatic microbiome in PDAC patients and repopulation of antibiotic ablated mice with the microbiome of tumor-bearing KPC mice or with *B. pseudolongum* accelerated oncogenesis	Microbial- targeted therapies as part of anti-tumor therapy	(19)

NA, not applicable.

Based on these results it can be envisioned that the above-mentioned microbiome modulating strategies increase therapeutic responses and survival of PDAC patients by lowering the abundance of CSC (properties). Fortunately, there are already ongoing randomized clinical trials that combine 16S rRNA gene analysis, FMT or probiotics along with chemotherapeutics and are listed in the review by Ciernikova et al. (57). The upcoming results may thus further substantiate the interrelationship of the host`s microbiome and tumor cells and provide the basis of novel therapeutic concepts of PDAC therapy.

## Discussion and Future Perspectives

**Figure 1:**
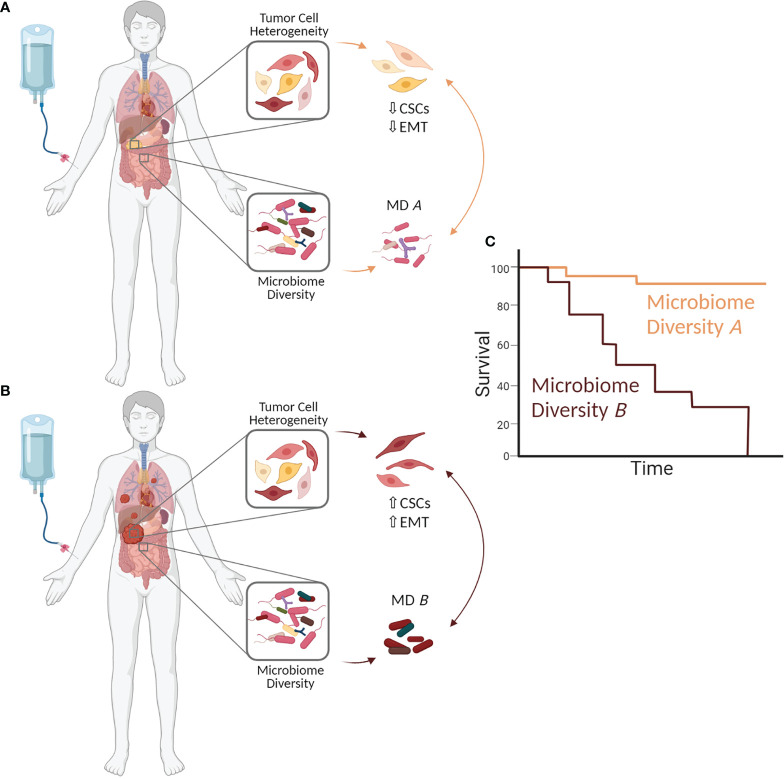
The microbiome impacts clonal heterogeneity of PDAC cells and thereby a patient´s outcome. Changes in the microbiome composition (in the tumor but also other body compartments) may influence Epithelial-Mesenchymal-Transition (EMT) and the abundance and phenotype of cancer stem cells (CSCs) in the tumor by generating optimal niche conditions and impacting their survival and expansion. This in turn impacts PDAC growth and progression as well as therapy responses and outcome of PDAC patients. **(A)** Microbiome diversity A (MD A) may prevent/control EMT induction and the enrichment of CSCs (CSC ↓), thereby inhibiting PDAC progression, drug resistance and **(C)** improving patient´s prognosis. **(B)** In contrast, microbiome diversity B (MD B) may induce EMT and increase the CSC potential within a tumor (CSC↑). As a consequence, a high CSC abundance promotes PDAC progression and drug resistance leading to **(C)** poor patient´s outcome. The figure was created with BioRender.com.

As summarized above and in the recently published review by Zhang et al. in this journal (8), the microbiome composition (in different body compartments) is considerably altered in PDAC patients compared to healthy individuals. This altered diversity may be a consequence of tumorigenesis, as the evolution of an inflammatory tumor microenvironment might promote bacterial translocation from the gut into the pancreas (8, 17, 21, 57). Besides, there is growing evidence that the microbiome is an important determinant of PDAC development and therapy response (8, 17, 21, 67–69). One mechanism by which the microbiome composition seems to drive PDAC progression and therapy resistance is promoting EMT. Importantly, EMT induction has been linked to the acquisition of CSC properties, and both EMT cells and CSC are characterized by profound drug resistance (30, 38–40). Considering these well-established interrelationships, it is reasonable to speculate that the abundance and plasticity of CSCs, and thereby intratumor heterogeneity in PDAC and patient´s outcome, are essentially influenced by the patient`s microbiome (**Figure 1**). This hypothesis is in line with studies supporting the fact that CSC properties can be gained or lost depending on the tumor microenvironment (24, 33–36). Since it is well known that an inflammatory microenvironment impacts the phenotype and genotype of tumor cells, it can be assumed that the altered composition of the gut as well as the tumor microbiome contribute to the inflammatory processes and thereby to the switch from a physiological (tumor suppressive) into an inflammatory (tumor promoting) tumor microenvironment. This in turn may induce EMT and CSC-properties in PDAC cells, e.g. by elevated levels of EMT/CSC inducing factors such as Transforming Growth Factor-beta1 or Tumor Necrosis Factor-alpha. Further, it cannot be ruled out that bacteria and their released factors directly induce EMT, as it could be demonstrated for *F. nucleatum*, and also promote the gain of CSC properties (56, 57, 61). A high abundance of CSCs in PDAC tissues could be related to PDAC dissemination, and with this progression and resistance to therapy (26, 31, 39, 41, 70–72). Hermann et al. (73) demonstrated that different CSC populations exist in PDAC and exhibit distinct functional capabilities. Thus, CD133+CXCR4+ CSCs were found to be particularly responsible for metastasis (73). Adding to the view of CSC heterogeneity in PDAC, own unpublished data indicate that PDAC cells can exhibit different CSC phenotypes that are characterized by distinct CSC marker expression (high Sox2 or high Nestin expressing CSCs) along with different migratory and invasive abilities. As a consequence, different metastasis patterns can be observed in a preclinical PDAC metastasis model (unpublished data). In line with this, Nestin was found to be upregulated in various human malignancies (74, 75) including PDAC, where it associated with an elevated liver metastatic potential of CSCs (31, 75). Considering this profound knowledge, we postulate that a more diverse microbiome composition, which was detected in LTS PDAC patients (21), might act in favor of a host defense by controlling the number and phenotype of CSCs in PDAC, resulting in a lower metastatic potential and less resistance towards chemotherapy (**Figure 1A**).

Accordingly, future studies are urgently needed to explore whether- and how a certain microbiome composition (e.g., those of LTS patients or *Fusobacteria*) influences intratumor heterogeneity through the gain and loss of CSC phenotypes, and in turn determines disease progression, therapy responses and survival of PDAC patients. Furthermore, since it is known that certain bacteria can increase the efficacy of therapy (18), the potential of microbiome modulation as an integral part of anti-cancer therapy needs to be further investigated. Given the fact that CSCs are mandatory for tumor initiation, novel therapeutic concepts aimed at their complete eradication. However, since the CSC pool can be constantly regenerated by conversion of non-CSC into CSCs, these strategies will likely ultimately fail. Instead, therapeutic strategies aiming to prevent or control CSCs may be more effective. Thus, the therapeutic enrichment of certain bacteria and/or restoring (physiological) microbiome diversity might be a promising strategy to effectively suppress the appearance, heterogeneity and survival of CSCs, thereby controlling disease progression and increasing the efficacy of therapeutics.

## Data Availability Statement

Publicly available datasets were analyzed in this study. This data can be found here: https://pubmed.ncbi.nlm.nih.gov.

## Author Contributions

Conceptualization, MB and L-MP. Supervision, SS and JB. Visualization, L-MP. Writing – original draft, MB, L-MP, and SS. Writing – review and editing, MB, L-MP, JB, and SS. All authors contributed to the article and approved the submitted version.

## Funding

This project and its publication were supported by the Research and Training Group 2501 on Translational Evolutionary Research (RTG 2501 TransEvo) funded by the Deutsche Forschungsgemeinschaft.

## Conflict of Interest

The authors declare that the research was conducted in the absence of any commercial or financial relationships that could be construed as a potential conflict of interest.

## Publisher’s Note

All claims expressed in this article are solely those of the authors and do not necessarily represent those of their affiliated organizations, or those of the publisher, the editors and the reviewers. Any product that may be evaluated in this article, or claim that may be made by its manufacturer, is not guaranteed or endorsed by the publisher.

## Abbreviations

CDD, cytidine deaminase; CSC, cancer stem cell; CTLA-4, cytotoxic T-lymphocyte-associated protein 4; EMT, Epithelial-Mesenchymal-Transition; FMT, fecal microbiota transplant; IPMNs, intraductal papillary mucinous neoplasms; LTS, long-term survivors; MET, Mesenchymal-Epithelial-Transition; PDAC, Pancreatic ductal adenocarcinoma; STS, short-term survivors.

## References

[1] Siegel RL Miller KD Fuchs HE Jemal A. Cancer Statistics, 2021. CA Cancer J Clin (2021) 71(1):7–33. doi: 10.3322/caac.21654 33433946

[2] Rahib L Smith BD Aizenberg R Rosenzweig AB Fleshman JM Matrisian LM. Projecting Cancer Incidence and Deaths to 2030: The Unexpected Burden of Thyroid, Liver, and Pancreas Cancers in the United States. Cancer Res (2014) 74(11):2913–21. doi: 10.1158/0008-5472.CAN-14-0155 24840647

[3] König J Hartel M Nies AT Martignoni ME Guo J Büchler MW. Expression and Localization of Human Multidrug Resistance Protein (ABCC) Family Members in Pancreatic Carcinoma. Int J Cancer (2005) 115(3):359–67. doi: 10.1002/ijc.20831 15688370

[4] Nath S Daneshvar K Roy LD Grover P Kidiyoor A Mosley L. MUC1 Induces Drug Resistance in Pancreatic Cancer Cells via Upregulation of Multidrug Resistance Genes. Oncogenesis (2013) 2(6):e51–9. doi: 10.1038/oncsis.2013.16 PMC3740301 23774063

[5] Wang Z Li Y Ahmad A Banerjee S Azmi AS Kong D. Pancreatic Cancer: Understanding and Overcoming Chemoresistance. Nat Rev Gastroenterol Hepatol (2011) 8(1):27–33. doi: 10.1038/nrgastro.2010.188 21102532

[6] Müerköster S Wegehenkel K Arlt A Witt M Sipos B Kruse ML. Tumor Stroma Interactions Induce Chemoresistance in Pancreatic Ductal Carcinoma Cells Involving Increased Secretion and Paracrine Effects of Nitric Oxide and Interleukin-1β. Cancer Res (2004) 64(4):1331–7. doi: 10.1158/0008-5472.CAN-03-1860 14973050

[7] Müerköster SS Werbing V Koch D Sipos B Ammerpohl O Kalthoff H. Role of Myofibroblasts in Innate Chemoresistance of Pancreatic Carcinoma - Epigenetic Downregulation of Caspases. Int J Cancer (2008) 123(8):1751–60. doi: 10.1002/ijc.23703 18649362

[8] Zhang W Zhang K Zhang P Zheng J Min C Li X. Research Progress of Pancreas-Related Microorganisms and Pancreatic Cancer. Front Oncol (2021) 10:1–12. doi: 10.3389/fonc.2020.604531 PMC7841623 33520714

[9] van Staalduinen J Baker D ten Dijke P van Dam H. Epithelial–mesenchymal-Transition-Inducing Transcription Factors: New Targets for Tackling Chemoresistance in Cancer? Oncogene (2018) 37(48):6195–211. doi: 10.2174/15680096113136660097 30002444

[10] Shang Y Cai X Fan D. Roles of Epithelial-Mesenchymal Transition in Cancer Drug Resistance. Curr Cancer Drug Targets (2014) 13(9):915–29. 10.2174/15680096113136660097 24168191

[11] Sui H Zhu L Deng W Li Q. Epithelial-Mesenchymal Transition and Drug Resistance: Role, Molecular Mechanisms, and Therapeutic Strategies. Oncol Res Treat (2014) 37(10):584–9. doi: 10.1159/000367802 25342509

[12] Sung PH Wen J Bang S Park S Si YS. CD44-Positive Cells Are Responsible for Gemcitabine Resistance in Pancreatic Cancer Cells. Int J Cancer (2009) 125(10):2323–31. doi: 10.1002/ijc.24573 19598259

[13] Ottinger S Klöppel A Rausch V Liu L Kallifatidis G Gross W. Targeting of Pancreatic and Prostate Cancer Stem Cell Characteristics by Crambe Crambe Marine Sponge Extract. Int J Cancer (2012) 130(7):1671–81. doi: 10.1002/ijc.26168 21544815

[14] Rajeshkumar NV Rasheed ZA García-García E López-Ríos F Fujiwara K Matsui WH. A Combination of DR5 Agonistic Monoclonal Antibody With Gemcitabine Targets Pancreatic Cancer Stem Cells and Results in Long-Term Disease Control in Human Pancreatic Cancer Model. Mol Cancer Ther (2010) 9(9):2582–92. doi: 10.1158/1535-7163.MCT-10-0370 PMC3700345 20660600

[15] Xia P Xu XY. PI3K/Akt/mTOR Signaling Pathway in Cancer Stem Cells: From Basic Research to Clinical Application. Am J Cancer Res (2015) 5(5):1602–9. PMC4497429 26175931

[16] Sharma N Nanta R Sharma J Gunewardena S Singh KP Shankar S. PI3K/AKT/mTOR and Sonic Hedgehog Pathways Cooperate Together to Inhibit Human Pancreatic Cancer Stem Cell Characteristics and Tumor Growth. Oncotarget (2015) 6(31):32039–60. doi: 10.18632/oncotarget.5055 PMC4741658 26451606

[17] Geller LT Barzily-Rokni M Danino T Jonas OH Shental N Nejman D. Potential Role of Intratumor Bacteria in Mediating Tumor Resistance to the Chemotherapeutic Drug Gemcitabine. Science (2017) 357(6356):1156–60. doi: 10.1126/science.aah5043 PMC5727343 28912244

[18] Vétizou M JM P Daillère R Lepage P Waldschmitt N Flament C. Anticancer Immunotherapy by CTLA-4 Blockade Relies on the Gut Microbiota. Science (2015) 350(6264):1079–84. doi: 10.1126/science.aad1329 PMC4721659 26541610

[19] Pushalkar S Hundeyin M Daley D Zambirinis CP Kurz E Mishra A. The Pancreatic Cancer Microbiome Promotes Oncogenesis by Induction of Innate and Adaptive Immune Suppression. Cancer Discov (2018) 8(4):403–16. doi: 10.1158/2159-8290.CD-17-1134 PMC6225783 29567829

[20] Iida N Dzutsev A Stewart CA Smith L Bouladoux N Weingarten RA. Commensal Bacteria Control Cancer Response to Therapy by Modulating the Tumor Microenvironment. Science (2013) 342(6161):967–70. doi: 10.1126/science.1240527 PMC6709532 24264989

[21] Riquelme E Zhang Y Zhang L Montiel M Zoltan M Dong W. Tumor Microbiome Diversity and Composition Influence Pancreatic Cancer Outcomes. Cell (2019) 178(4):795–806.e12. doi: 10.1016/j.cell.2019.07.008 31398337 PMC7288240

[22] Nieto MA Huang RYYJ Jackson RAA Thiery JPP. Emt: 2016. Cell (2016) 166(1):21–45. doi: 10.1016/j.cell.2016.06.028 27368099

[23] Andriani F Bertolini G Facchinetti F Baldoli E Moro M Casalini P. Conversion to Stem-Cell State in Response to Microenvironmental Cues Is Regulated by Balance Between Epithelial and Mesenchymal Features in Lung Cancer Cells. Mol Oncol (2016) 10(2):253–71. doi: 10.1016/j.molonc.2015.10.002 PMC5528953 26514616

[24] Schwitalla S Fingerle AA Cammareri P Nebelsiek T Göktuna SI Ziegler PK. Intestinal Tumorigenesis Initiated by Dedifferentiation and Acquisition of Stem-Cell-Like Properties. Cell (2013) 152(1–2):25–38. doi: 10.1016/j.cell.2012.12.012 23273993

[25] Mani SA Guo W Liao M Eaton EN Zhou AY Brooks M. EMT Creates Cells With the Properties of Stem Cells. Cell (2008) 133(4):704–15. doi: 10.1016/j.cell.2008.03.027 PMC2728032 18485877

[26] Batlle E Clevers H. Cancer Stem Cells Revisited. Nat Med (2017) 23(10):1124–34. doi: 10.1038/nm.4409 28985214

[27] Kreso A Dick JE. Evolution of the Cancer Stem Cell Model. Cell Stem Cell (2014) 14(3):275–91. doi: 10.1016/j.stem.2014.02.006 24607403

[28] Greaves M. Cancer Stem Cells as “Units of Selection”. Evol Appl (2013) 6(1):102–8. doi: 10.1111/eva.12017 PMC3567475 23396760

[29] Quail D F Taylor M J Postovit L-M. Microenvironmental Regulation of Cancer Stem Cell Phenotypes. Curr Stem Cell Res Ther (2012) 7(3):197–216. doi: 10.2174/157488812799859838 22329582

[30] Lambert AW Pattabiraman DR Weinberg RA. Emerging Biological Principles of Metastasis. Cell (2017) 168(4):670–91. doi: 10.1016/j.cell.2016.11.037 PMC5308465 28187288

[31] Knaack H Lenk L Philipp L-M Miarka L Rahn S Viol F. Liver Metastasis of Pancreatic Cancer: The Hepatic Microenvironment Impacts Differentiation and Self-Renewal Capacity of Pancreatic Ductal Epithelial Cells. Oncotarget (2018) 9(60):31771–86. doi: 10.18632/oncotarget.25884 PMC6114965 30167093

[32] Fulawka L Donizy P Halon A. Cancer Stem Cells–the Current Status of an Old Concept: Literature Review and Clinical Approaches. Biol Res (2014) 47:66. doi: 10.1186/0717-6287-47-66 25723910 PMC4335556

[33] Nallasamy P Nimmakayala RK Karmakar S Leon F Seshacharyulu P Lakshmanan I. Pancreatic Tumor Microenvironment Factor Promotes Cancer Stemness *via* SPP1-CD44 Axis. Gastroenterology (2021) S0016–5085(21)03402-8. doi: 10.1053/j.gastro.2021.08.023. PMC10069715 34418441

[34] O’Leary DP O’Leary E Foley N Cotter TG Wang JH Redmond HP. Effects of Surgery on the Cancer Stem Cell Niche. Eur J Surg Oncol (2016) 42(3):319–25. doi: 10.1016/j.ejso.2015.12.008 26810247

[35] Pozza ED Dando I Biondani G Brandi J Costanzo C Zoratti E. Pancreatic Ductal Adenocarcinoma Cell Lines Display a Plastic Ability to Bi-Directionally Convert Into Cancer Stem Cells. Int J Oncol (2015) 46(3):1099–108. doi: 10.3892/ijo.2014.2796 25502497

[36] Chaffer CL Brueckmann I Scheel C Kaestli AJ Wiggins PA Rodrigues LO. Normal and Neoplastic Nonstem Cells can Spontaneously Convert to a Stem-Like State. Proc Natl Acad Sci USA (2011) 108(19):7950–5. doi: 10.1073/pnas.1102454108 PMC3093533 21498687

[37] Crawford HC Pasca di Magliano M Banerjee S. Signaling Networks That Control Cellular Plasticity in Pancreatic Tumorigenesis, Progression, and Metastasis. Gastroenterology (2019) 156(7):2073–84. doi: 10.1053/j.gastro.2018.12.042 PMC6545585 30716326

[38] Najafi M Mortezaee K Majidpoor J. Cancer Stem Cell (CSC) Resistance Drivers. Life Sci (2019) 234:116781. doi: 10.1016/j.lfs.2019.116781 31430455

[39] Valle S Martin-Hijano L Alcalá S Alonso-Nocelo M Sainz B. The Ever-Evolving Concept of the Cancer Stem Cell in Pancreatic Cancer. Cancers (Basel) (2018) 10(2):33. doi: 10.3390/cancers10020033 PMC5836065 29373514

[40] Steinbichler TB Dudás J Skvortsov S Ganswindt U Riechelmann H Skvortsova II. Therapy Resistance Mediated by Cancer Stem Cells. Semin Cancer Biol (2018) 53:156–67. doi: 10.1016/j.semcancer.2018.11.006 30471331

[41] Aponte PM Caicedo A. Stemness in Cancer: Stem Cells, Cancer Stem Cells, and Their Microenvironment. Stem Cells Int (2017) 2017:5619472. doi: 10.1155/2017/5619472 28473858 PMC5394399

[42] Heiler S Wang Z Zöller M. Pancreatic Cancer Stem Cell Markers and Exosomes - The Incentive Push. World J Gastroenterol (2016) 22(26):5971–6007. doi: 10.3748/wjg.v22.i26.5971 27468191 PMC4948278

[43] Patil K Khan FB Akhtar S Ahmad A Uddin S. The Plasticity of Pancreatic Cancer Stem Cells: Implications in Therapeutic Resistance. Cancer Metastasis Rev (2021). doi: 10.1007/s10555-021-09979-x. Online ahead of print PMC8556195 34453639

[44] Eckburg PB Bik EM Bernstein CN Purdom E Dethlefsen L Sargent M. Microbiology: Diversity of the Human Intestinal Microbial Flora. Science (2005) 308(5728):1635–8. doi: 10.1126/science.1110591 PMC1395357 15831718

[45] Ley RE Peterson DA Gordon JI. Ecological and Evolutionary Forces Shaping Microbial Diversity in the Human Intestine. Cell (2006) 124(4):837–48. doi: 10.1016/j.cell.2006.02.017 16497592

[46] Arrieta MC Arévalo A Stiemsma L Dimitriu P Chico ME Loor S. Associations Between Infant Fungal and Bacterial Dysbiosis and Childhood Atopic Wheeze in a Nonindustrialized Setting. J Allergy Clin Immunol (2018) 142(2):424–34.e10. doi: 10.1016/j.jaci.2017.08.041 29241587 PMC6075469

[47] Gevers D Kugathasan S Denson LA Vázquez-Baeza Y Van Treuren W Ren B. The Treatment-Naive Microbiome in New-Onset Crohn’s Disease. Cell Host Microbe (2014) 15(3):382–92. doi: 10.1016/j.chom.2014.02.005 PMC4059512 24629344

[48] Harsch I Konturek P. The Role of Gut Microbiota in Obesity and Type 2 and Type 1 Diabetes Mellitus: New Insights Into “Old” Diseases. Med Sci (2018) 6(2):32. doi: 10.3390/medsci6020032 PMC6024804 29673211

[49] Tang WHW Li DY Hazen SL. Dietary Metabolism, the Gut Microbiome, and Heart Failure. Nat Rev Cardiol (2019) 16:137–54. Nature Publishing Group. doi: 10.1038/s41569-018-0108-7 PMC6377322 30410105

[50] Dzutsev A Goldszmid RS Viaud S Zitvogel L Trinchieri G. The Role of the Microbiota in Inflammation, Carcinogenesis, and Cancer Therapy. Eur J Immunol (2015) 45(1):17–31. doi: 10.1002/eji.201444972 25328099

[51] Nishida A Inoue R Inatomi O Bamba S Naito Y Andoh A. Gut Microbiota in the Pathogenesis of Inflammatory Bowel Disease. Clin J Gastroenterol (2018) 11(1):1–10. doi: 10.1007/s12328-017-0813-5 29285689

[52] Maekawa T Fukaya R Takamatsu S Itoyama S Fukuoka T Yamada M. Possible Involvement of Enterococcus Infection in the Pathogenesis of Chronic Pancreatitis and Cancer. Biochem Biophys Res Commun (2018) 506(4):962–9. doi: 10.1016/j.bbrc.2018.10.169 30401562

[53] Guo Y Liu W Wu J. Helicobacter Pylori Infection and Pancreatic Cancer Risk: A Meta-Analysis. J Cancer Res Ther (2016) 12(8):C229–32. doi: 10.4103/0973-1482.200744 28230023

[54] Padoan A Plebani M Basso D. Inflammation and Pancreatic Cancer: Focus on Metabolism, Cytokines, and Immunity. Int J Mol Sci (2019) 20(3):676. doi: 10.3390/ijms20030676 PMC6387440 30764482

[55] Hofman P Vouret-Craviari V. Microbes-Induced EMT at the Crossroad of Inflammation and Cancer. Gut Microbes (2012) 3(3):176–85. doi: 10.4161/gmic.20288 PMC3427211 22572828

[56] Gaiser RA Halimi A Alkharaan H Lu L Davanian H Healy K. Enrichment of Oral Microbiota in Early Cystic Precursors to Invasive Pancreatic Cancer. Gut (2019) 68(12):2186–94. doi: 10.1136/gutjnl-2018-317458 PMC6872446 30872392

[57] Ciernikova S Mego M Novisedlakova M Cholujova D Stevurkova V. The Emerging Role of Microbiota and Microbiome in Pancreatic Ductal Adenocarcinoma. Biomedicines (2020) 8(12):1–21. doi: 10.3390/biomedicines8120565 PMC7761686 33287196

[58] Mitsuhashi K Nosho K Sukawa Y Matsunaga Y Ito M Kurihara H. Association of Fusobacterium Species in Pancreatic Cancer Tissues With Molecular Features and Prognosis. Oncotarget (2015) 6(9):7209–20. doi: 10.18632/oncotarget.3109 PMC4466679 25797243

[59] Rogers MB Aveson V Firek B Yeh A Brooks B Brower-Sinning R. Disturbances of the Perioperative Microbiome Across Multiple Body Sites in Patients Undergoing Pancreaticoduodenectomy. Pancreas (2017) 46(2):260–7. doi: 10.1097/MPA.0000000000000726 PMC5235958 27846140

[60] Tada K Ohta M Hidano S Watanabe K Hirashita T Oshima Y. Fucosyltransferase 8 Plays a Crucial Role in the Invasion and Metastasis of Pancreatic Ductal Adenocarcinoma. Surg Today (2020) 50(7):767–77. doi: 10.1007/s00595-019-01953-z 31950256

[61] Hosein AN Brekken RA Maitra A. Pancreatic Cancer Stroma: An Update on Therapeutic Targeting Strategies. Nat Rev Gastroenterol Hepatol (2020) 17(8):487–505. doi: 10.1038/s41575-020-0300-1 32393771 PMC8284850

[62] Vergara D Simeone P Damato M Maffia M Lanuti P Trerotola M. The Cancer Microbiota: EMT and Inflammation as Shared Molecular Mechanisms Associated With Plasticity and Progression. J Oncol (2019) 2019:1253727. doi: 10.1155/2019/1253727 31772577 PMC6854237

[63] Sivan A Corrales L Hubert N Williams JB Aquino-Michaels K Earley ZM. Commensal Bifidobacterium Promotes Antitumor Immunity and Facilitates Anti-PD-L1 Efficacy. Science (2015) 350(6264):1084–9. doi: 10.1126/science.aac4255 PMC4873287 26541606

[64] Matson V Fessler J Bao R Chongsuwat T Zha Y Alegre ML. The Commensal Microbiome Is Associated With Anti-PD-1 Efficacy in Metastatic Melanoma Patients. Science (2018) 359(6371):104–8. doi: 10.1126/science.aao3290 PMC6707353 29302014

[65] Mendez R Kesh K Arora N Di ML McAllister F Merchant N. Microbial Dysbiosis and Polyamine Metabolism as Predictive Markers for Early Detection of Pancreatic Cancer. Carcinogenesis (2020) 41(5):561–70. doi: 10.1093/carcin/bgz116 PMC7350554 31369062

[66] Leinwand JC Miller G. Microbes as Biomarkers and Targets in Pancreatic Cancer. Nat Rev Clin Oncol (2019) 16(11):665–6. doi: 10.1038/s41571-019-0276-3 31530941

[67] Huang J Jiang Z Wang Y Fan X Cai J Yao X. Modulation of Gut Microbiota to Overcome Resistance to Immune Checkpoint Blockade in Cancer Immunotherapy. Curr Opin Pharmacol (2020) 54:1–10. doi: 10.1016/j.coph.2020.06.004 32619934

[68] Thomas RM Jobin C. Microbiota in Pancreatic Health and Disease: The Next Frontier in Microbiome Research. Nat Rev Gastroenterol Hepatol (2020) 17(1):53–64. doi: 10.1038/s41575-019-0242-7 31811279

[69] Wei MY Shi S Liang C Meng QC Hua J Zhang YY. The Microbiota and Microbiome in Pancreatic Cancer: More Influential Than Expected. Mol Cancer (2019) 18(1):1–15. doi: 10.1186/s12943-019-1008-0 31109338 PMC6526613

[70] Fabian A Stegner S Miarka L Zimmermann J Lenk L Rahn S. Metastasis of Pancreatic Cancer: An Uninflamed Liver Micromilieu Controls Cell Growth and Cancer Stem Cell Properties by Oxidative Phosphorylation in Pancreatic Ductal Epithelial Cells. Cancer Lett (2019) 453:95–106. doi: 10.1016/j.canlet.2019.03.039 30930235

[71] Dando I Dalla Pozza E Biondani G Cordani M Palmieri M Donadelli M. The Metabolic Landscape of Cancer Stem Cells. IUBMB Life (2015) 67(9):687–93. doi: 10.1002/iub.1426 26337609

[72] Hanahan D Weinberg RA. Hallmarks of Cancer: The Next Generation. Cell (2011) 144(5):646–74. doi: 10.1016/j.cell.2011.02.013 21376230

[73] Hermann PC Huber SL Herrler T Aicher A Ellwart JW Guba M. Distinct Populations of Cancer Stem Cells Determine Tumor Growth and Metastatic Activity in Human Pancreatic Cancer. Cell Stem Cell (2007) 1(3):313–23. doi: 10.1016/j.stem.2007.06.002 18371365

[74] Neradil J Veselska R. Nestin as a Marker of Cancer Stem Cells. Cancer Sci (2015) 106(7):803–11. doi: 10.1111/cas.12691 PMC4520630 25940879

[75] Matsuda Y Naito Z Kawahara K Nakazawa N Korc M Ishiwata T. Nestin Is a Novel Target for Suppressing Pancreatic Cancer Cell Migration, Invasion and Metastasis. Cancer Biol Ther (2011) 11(5):512–23. doi: 10.4161/cbt.11.5.14673 PMC3230315 21258211

